# Swallow Detection with Acoustics and Accelerometric-Based Wearable Technology: A Scoping Review

**DOI:** 10.3390/ijerph20010170

**Published:** 2022-12-22

**Authors:** Bryan Pak-Hei So, Tim Tin-Chun Chan, Liangchao Liu, Calvin Chi-Kong Yip, Hyo-Jung Lim, Wing-Kai Lam, Duo Wai-Chi Wong, Daphne Sze Ki Cheung, James Chung-Wai Cheung

**Affiliations:** 1 Department of Biomedical Engineering, Faculty of Engineering, The Hong Kong Polytechnic University, Hong Kong; 2 Physical Education Department, University of International Business and Economics, Beijing 100029, China; 3 School of Medical and Health Sciences, Tung Wah College, Hong Kong; 4 Sports Information and External Affairs Centre, Hong Kong Sports Institute, Hong Kong; 5 School of Nursing, The Hong Kong Polytechnic University, Hong Kong; 6 Research Institute of Smart Ageing, The Hong Kong Polytechnic University, Hong Kong

**Keywords:** dysphagia, deglutition disorder, eating disorder, otorhinolaryngology, mHealth

## Abstract

Swallowing disorders, especially dysphagia, might lead to malnutrition and dehydration and could potentially lead to fatal aspiration. Benchmark swallowing assessments, such as videofluoroscopy or endoscopy, are expensive and invasive. Wearable technologies using acoustics and accelerometric sensors could offer opportunities for accessible and home-based long-term assessment. Identifying valid swallow events is the first step before enabling the technology for clinical applications. The objective of this review is to summarize the evidence of using acoustics-based and accelerometric-based wearable technology for swallow detection, in addition to their configurations, modeling, and assessment protocols. Two authors independently searched electronic databases, including PubMed, Web of Science, and CINAHL. Eleven (*n* = 11) articles were eligible for review. In addition to swallowing events, non-swallowing events were also recognized by dry (saliva) swallowing, reading, yawning, etc., while some attempted to classify the types of swallowed foods. Only about half of the studies reported that the device attained an accuracy level of >90%, while a few studies reported poor performance with an accuracy of <60%. The reviewed articles were at high risk of bias because of the small sample size and imbalanced class size problem. There was high heterogeneity in assessment protocol that calls for standardization for swallowing, dry-swallowing and non-swallowing tasks. There is a need to improve the current wearable technology and the credibility of relevant research for accurate swallowing detection before translating into clinical screening for dysphagia and other swallowing disorders.

## 1. Introduction

Swallowing is a natural yet essential part of our daily life. Human performs spontaneous swallowing (saliva and food/drink) 0.98 times per minute on average [1]. With different definitions and measurement techniques, Lear et al. [2] suggested that humans swallow approximately 200 to 1000 times a day, while Rudney et al. [3] reported that spontaneous swallows are performed by healthy humans 18 to 400 times per hour. However, some people may have difficulty swallowing, especially aged people or people with chronic conditions. Swallowing difficulty is also termed dysphagia, in which dysphagic individuals have problems chewing and swallowing food or liquids, experience pain during swallowing, or even be unable to swallow. Notably, the bolus may enter the airway and lungs, leading to aspiration pneumonia, which is fatal but clinically silent [4]. Dysphagia is generally chronic but deteriorates with the worsening of cognition and functions in the progression of dementia or other neurological disorders [5,6]. Therefore, continuous monitoring or assessment could be necessary to identify the stage at high risk of choking or aspiration for timely management and rehabilitation [7,8]. In addition, dysphagia patients may be reluctant to eat due to the fear of choking, pain, or difficulty that causes malnutrition, dehydration, depression, and anorexia [9]. More than one-third of older adults reported dysphagia or swallowing disorders during their lifetime, which were associated with stroke, diabetes, Parkinson’s, and Alzheimer’s disease [10,11]. Howden [12] and Ney et al. [13] reported that the prevalence of dysphagia could be 22% and 40% for seniors aged over 50 and 60, respectively. A recent survey reported that swallowing difficulty was reported in one in every six adults, and some of them might not seek medical care [14].

Swallowing assessment or monitoring is imperative to facilitate early diagnosis, management, or rehabilitation to reduce mortality and improve the quality of life for dysphagia individuals. Nowadays, the Videofluoroscopic Swallowing Study (VFSS) and Fiberoptic Endoscopic Evaluation of Swallowing (FEES) are golden standards for instrumented assessment [15]. VFSS applies a dynamic fluoroscopic imaging technique to visualize the detailed swallowing process in oral, pharyngeal, laryngeal, and oesophageal regions in real-time [16]. For FEES, practitioners inspect the postural maneuvers of the nasal structures when the patients speak, eat, and breathe using an endoscope [17]. However, VFSS and FEES are expensive, cause discomfort and risks to the patients, and can only be conducted occasionally.

Non-instrumental bedside assessments for swallowing are alternatives to compromise cost and test frequency that could be readily adopted in nursing homes or care homes by an occupational therapist or speech therapist. A standard bedside screening process involves anamnesis assessment, morphodynamical evaluation, gustative function with specific stimulation test, and the oral feeding test [18]. Other related tests include the 3-ounce water swallowing test [19], cough reflex test [20], and cervical auscultation, which uses a stethoscope to amplify and listen to the swallowing sound [21]. Most of these instruments lacked sensitivity and predictive strength and poor reproducibility and consistency in the protocols [21,22] but could be routinely conducted for initial screening of swallowing functions [23].

Cervical auscultation refers to the measurement of sound or vibration of the throat for swallowing assessment, which is traditionally conducted by physicians using a stethoscope [24]. Wearable technology, such as accelerometry, acoustics, and electromyogram, could be more robust to facilitate non-invasive and non-ionizing, continuous monitoring or screening with less cost. Swallowing accelerometry monitors the translation of vibration through the aerodigestive tract and hyoid bone kinetics during swallowing [25]. The acoustic technique uses an inexpensive microphone to record swallowing sounds and may sometimes integrate with the accelerometry approach [26]. Takahashi et al. [27] could be among the pioneers that systematically reviewed and evaluated acoustic methods for the detection of swallowing sounds, while Taveira et al. [28] reviewed and compared the diagnostic validity of swallowing-sound-based methods to videofluoroscopy. Thereafter, more developments have been conducted using multimodal sensors, advanced data processing techniques and machine learning models.

Dysphagia could be the most significant swallowing problem, but eating behavior disorders and nutrition problems might also require long-term swallowing assessment/monitoring. Acoustic-based and accelerometric-based approaches are currently the most promising technique and well-researched areas to standardize and formalize to be a clinical screening instrument and protocol. To this end, we aim to review preclinical study articles that evaluated the accuracy of acoustics or/and accelerometric-based instruments in identifying swallowing events/scenarios of healthy individuals. The goal of this review is to summarize evidence on the techniques, protocols, and performances on the assessment of “healthy swallows” (i.e., delimited non-healthy swallows from the scope of this review) because it is important to establish the baseline evidence for “healthy swallows” before those on non-healthy swallows (e.g., dysphagia) could be credited. To achieve the goal, the review questions of this study are as follows:

- What acoustic or/and accelerometric-based sensors were used for swallowing detection, and how where were they configurated?
- What were the protocols and procedures to apply those sensors for swallowing detection?
- How was the collected signal processed and extracted that manifested the swallowing event?
- How accurate were these techniques and protocols in identifying swallowing events or classes?

## 2. Materials and Methods

The scoping review was conducted according to the JBI protocol recommendation [29]. A literature search was performed on electronic databases, including PubMed, Web of Science, and CINAHL (via EBSCOHost). The search was conducted using a combination of keywords on areas related to dysphagia, sensors, and outcome measures. Keywords for dysphagia included “Dysphagia”, “dysphag*”, “deglutition”, or “swallowing”. Keywords for sensors included “accelero*”, “acoustic”, “high resolution cervical auscultation”, “MMG”, “mechanomyo*”, “vibration”, “sonic”, “motion”, “microphone”, or “sound”. Keywords for parameters included “confusion matrix”, “sensitivity”, “specificity”, “accuracy”. “AUC”, “area under curve”, “positive predictive value”. “PPV”, “negative predictive value”, “NPV”, “F1 score”, “F1-score”, “recall”, or “precision”.

The literature search was limited to original research articles written in English. The inclusion criteria included the evaluation study of swallowing detection instruments that applied either accelerometry or/and acoustics or fusion with other technologies. The evaluation shall be conducted on human subjects to detect swallowing or to classify the swallowed constituents nonmanually. The outcome measures shall involve accuracy-related metrics (such as precision and recall, etc.). According to the scope of our review to summarize evidence on the baseline (i.e., healthy swallows), articles that tested on non-healthy participants were excluded, including dysphagia, coughing, stroke, and aspirated individuals. Nevertheless, articles would not be excluded regardless of the level of body mass index (BMI) if the subjects were recognized as “healthy subjects”. Studies were also excluded if their primary goals were not the evaluation of instruments, for example, applying the instrument to evaluate the effects of interventions. Furthermore, studies were excluded if they did not clarify the investigation on “swallowing”, such as those that only mentioned food intake or chewing.

The literature search was conducted on 1 April 2022 by two independent authors (B.P.-H.S. and D.W.-C.W.). The first author further conducted the screening of abstracts and full texts, which was reviewed by the second author. Any disagreement was resolved by seeking consensus with the corresponding author (J.W.-C.W.). Data reported in the individual reports around the three primary themes were extracted for analysis: (1) Instrument configuration; (2) swallowing tasks or assessment protocols for the instrument evaluation; (3) settings and performances of the classification.

## 3. Results

### 3.1. Search Results

**Figure 1:**
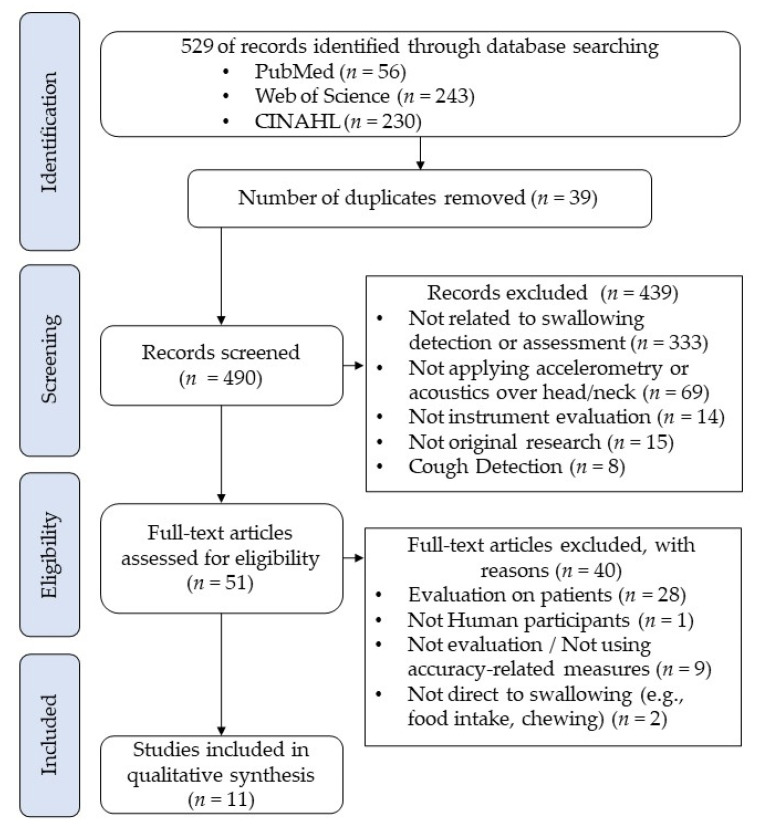
PRISMA flowchart of systematic search and screening.

The search and screening process is illustrated in Figure 1. There was no disagreement among authors in the selection of studies. The initial search yielded 529 records, and 490 articles were identified after removing 39 duplicates. The first level of screening on the title and abstract excluded 439 articles because of irrelevancy to the swallowing detection (*n* = 333); not utilizing accelerometry and acoustics sensors on the head-neck region (*n* = 69); not conducting instrument evaluation (*n* = 14); not original research articles (*n* = 15); and dedicated to cough detection instead of swallowing (*n* = 8). Screening on the full texts further excluded 40 articles with reasons including evaluation of non-healthy patients, such as dysphagia, stroke, aspiration, Parkinson’s disease (*n* = 28); evaluation not on Human subjects; not conducting instrument evaluation or not including any accuracy-related outcome measures (*n* = 9); not direct to swallowing assessment, such as food intake, and chewing (*n* = 2). Eventually, there were 11 articles eligible for the review [30,31,32,33,34,35,36,37,38,39,40]. It shall be noted that three articles came from the same research team [33,37,38], whilst two other articles were also presented by another research team [36,39].

### 3.2. Instrument Configuration

Among the 11 eligible articles, five of them utilized only acoustics (microphone) [32,33,34,35,40], one utilized only accelerometers in the instrument [39], and five applied a multimodal system [30,31,36,37,38]. However, two articles on multimodal systems did not fully describe the modalities other than acoustics [30,37]. Other multimodal systems involved surface electromyography (sEMG), mechanomyography (MMG), and airflow pressure sensor.

As shown in Table 1, a single microphone for detecting swallowing sounds appeared in three articles [32,35,40]. Skowronski et al. [40] made use of a miniature surface-mounted microphone and characterized the signal using Human Factor Cepstral Coefficients [41], which was originally used for automatic speech recognition. Bi et al. [32] developed the “AutoDietary” system using a throat microphone. The system also displayed the food type recognition results for the users for personal health management. Kurihara et al. [35] customized the device by attaching a bi-directional electret condenser microphone on the ends of an air tube to detect the swallowing microphone through the pressure propagation along the air tube. Two studies employed two microphones but with different principles [33,34]. The major laryngeal microphone was used to record the swallowing sound directly in both cases. On the one hand, Fukuike et al. [34] further improved the system accuracy by adding a condenser microphone on the nostril. On the other hand, Fontana et al. [33] used the condenser microphone to detect the swallowing sound in the subsonic range. Additionally, Amft and Troster [31] integrated a stethoscope microphone with sEMG of the cricopharyngeus muscle to recognize swallowing. They also presented separate analyses on dietary movement activity and chewing activity recognition using other sensors [31].

Accelerometry measurements were presented in three papers [30] and two incorporated in the multimodal system [36,39]. Afkari [30] implemented a tri-modal system using miniature accelerometers, sEMG, and omnidirectional electret microphone, while Lee et al. [36] targeted the nasal airflow measured by a pressure transducer and the submental MMG developed previously [42]. All these devices made use of biaxial accelerometers aligned in anterior-posterior and superior-inferior directions [30,36,39].

There were variations in the locations of the sensors, which may depend on the types and the suspension methods. Although few studies vaguely mentioned that the sensors shall be attached over the laryngopharynx, thyroid cartilage and cricoid cartilage were two anatomical landmarks highlighted [30,36,39,40]. The sensors could be glued or taped to the throat surface [30,39], collared [31], or in the form of a necklace [33,34,35,36].

### 3.3. Assessment Protocol for Swallowing

Since swallowing is a continuous process, segmenting a time frame to stamp the swallowing episode is essential to define the “sample counts” for evaluating accuracy. The episode stamping method could be classified as event-based or episode-based. Two studies attempted both event-based and episode-based approaches for the evaluation [37,38]. For the other studies, five [30,32,34,35,39] adopted the event-based approach, and four [31,33,36,40] adopted the episode-based approach, respectively.

For event-based stamping, the conditions were controlled, and the researchers instructed the participants to perform one maneuver at a time, in which the event could be easily labeled for a period. For the epoch-based approach, the participants were often free to conduct a series of activities at each time. Then, the time was sliced into several non-overlapping time units (epochs) by algorithms or data processing techniques and was then manually labeled by revisiting the videotape. Alternatively, participants might be asked to press a button or pedal during their swallowing process for labeling [33,34].

The swallowing protocol could be broadly classified as non-swallowing maneuvers and swallowing maneuvers, while some studies attempted to have a fine-grained classification within these two categories (Table 2). For non-swallowing, the dry swallow was referred to as saliva swallowing [30,39,40], while assessing non-swallowing through silence or talking was often implemented through an epoch-based approach (detailed in the next paragraph) [31,33,37,38]. Some studies investigated different types of throat movements as non-swallowing events, including yawning, coughing, sighing, sniffing, throat clearing, gargling, speech, and tongue moving [34,40]. Besides, it shall be noted that Fukuike et al. [34] considered sipping tea as a non-swallowing maneuver. On the other hand, there was no consensus on the kinds of food to prompt swallowing events. For the epoch-based approach, participants were asked to take a meal with a variety of food without controlling participants to eat one kind of food at a time during the data collection. Besides, drinking water appeared in most of the articles [30,31,32,33,36,39,40], while yogurt was the most famous semifluid food [31,33,37]. For solid food, bread, crackers, cookies, pizza, sandwiches, fruit, and peanuts were some examples considered [31,32,33,37].

### 3.4. Segmentation and Feature Extraction Strategy

Researchers had to identify whether a swallowing event happened within a time frame because of the continuous nature of swallowing, as shown in Table 3. Two studies manually segmented the time window [30,40], while four studies specified the duration of the segmented time window, ranging from 200 ms to 1.5 s [31,33,36,37]. Fukuike et al. [34], Kurihara et al. [35], and Sejdic et al. [39] utilized the semblable wave period, template matching, and minimum description length-based segmentation, respectively. Two studies accounted for randomized sampling concepts in the segmentation process, including the Hidden Markov Model (HMM) conducted by Bi et al. [32] and the grid search conducted by Sazonov et al. [38].

For the feature extraction strategy, four studies exploited the time-domain raw signals for classification [30,33,34,39], while one made use of the frequency-domain raw signals [38]. Predetermined features were computed for analysis in three articles [32,35,36]. For example, Amft and Troster [31] considered and fused the spectral features (band energy, autocorrelation coefficient, and energy) and EMG features (total and maximum). Three studies performed some data reduction processes and established specific index parameters before the classification process [31,37,40], such as using Principal Component Analysis (PCA).

### 3.5. Classification and Performance

Depending on the nature of the classification (i.e., swallowing vs. non-swallowing or classification of different food types) and the stamping approach (i.e., event-based vs. epoch-based), studies might apply different classification approaches. In order to classify/identify the swallowing event, three studies applied a threshold-based approach [30,33,34], while others implemented statistical or machine learning models [31,32,35,36,37,38,39,40]. These models included logistic regression, decision tree, Gaussian Mixture Model (GMM), Support Vector Machine (SVM), Artificial Neural Network (ANN), etc.

For the threshold-based approach, a swallowing event was often recognized whenever the collected signal exceeded a predefined threshold value for more than a certain time. Nevertheless, the cut-off level or time range was not adequately justified in the papers, and most of them were empirical. Amft and Troster [31] applied compared acoustics, accelerometry, and EMG data with a set of reference voltages and integrated them by a logic gate (AND) but without justifying the source of the reference set. Fontana et al. [33] established individualized threshold levels based on the collected signal during a reading task. They also suggested that the time range threshold shall be 0.6 s [33], which was an estimated time for a complete swallow [38]. On the other hand, Fukuike et al. [34] decided to use twice the mean baseline as the threshold level, and a recognized event shall last longer than 0.35 s.

For the evaluation of classification performance, accuracy, sensitivity, specificity, and positive predictive value (PPV) are common evaluation metrics. Sensitivity and PPV are also sometimes termed precision and recall from the perspective of information retrieval in the field of data science [43]. In our reviewed articles, sensitivity represented the proportion of recognizing a swallowing event/class when that event/class did occur, while specificity was the proportion of recognizing not a swallowing event/class when that event/class had not occurred. Accuracy is the ratio of correct classifications over the total number of tests. Besides, one study [32] supplemented the receiver operating characteristics (ROC) curve to demonstrate the discrimination capacity.

As a rule of thumb, classifiers required an independent dataset for training and testing (model evaluation) to better evaluate the generalizing capability. Sejdic et al. [39] evaluated the model using both synthetic tests and real swallowing signals. Despite a different number of folds, most of the model-based classifiers applied k-fold cross-validation, while Kurihara et al. [35] adopted a leave-one-out approach. In addition, Lee et al. [36] calculated the accuracy metrics based on a bootstrapping augmentation after a 10-fold cross-validation of the model to account for the unbalanced class sizes.

The 11 reviewed articles involved 15 classifiers in our data synthesis (Table 4). There was a high variation in accuracy level among studies, ranging from 68.2% to 96.8%. We did not find any observable association between accuracy and the type of classifiers. Only about half (6/11) of the studies reached a satisfactory level of accuracy (>90%). Some studies had a classification performance as unreliable as a random guess (40–60%). Besides, despite that the accuracy metric of the review articles is generally satisfactory, the outcomes of other metrics (such as sensitivity, specificity, and PPV) could be quite different between studies. For example, Makeyev et al. [37] attained 44% sensitivity and 99% specificity in their epoch-based SVM model. Amft and Troster [31] got 20% positive predictive value and 68% sensitivity in their classification method using the agreement of detectors. The reason could be due to the problem of imbalanced class size, especially for epoch-based approaches.

## 4. Discussion

In summary, acoustics-based and accelerometric-based sensors have been used to identify swallowing events from non-swallowing events, which could be manifested by dry (saliva) swallowing, reading, yawning, etc. For swallowing events, attempts had been made to classify the type of food swallowed, such as solid versus liquid food and liquid with different viscosity (thickness). The identification strategy could be event-based or epoch-based. The former was often achieved by instructing the swallowing action and labeled manually by observation, while participants in the latter were asked to speak or to eat freely. The participants then pressed a button/pedal when they were performing the swallowing maneuver. There were variations in the sensor placement and configurations, which could be dependent on the selection and design of the sensor/instrument. However, our review showed that the overall successful recognition (or classification) rate was not satisfactory. About half of the studies attained an accuracy level >90%, while a few studies had poor performance with an accuracy of <60% on classifying swallowing actions. A correct classification of swallowing actions is essential before putting forward on non-healthy subjects. Otherwise, the system may not be able to distinguish signal deviation between swallowing actions or healthy versus non-healthy (e.g., dysphagia). Several articles adopted a threshold-based approach in classification but without adequate justification for the cut-off values. There was also heterogeneity in the segmentation of the swallowing period and feature extraction strategy. Future studies may consider deep learning models to allow self-extracted optimal windowing frames and features.

We challenge the credibility of the reviewed articles, both in terms of external and internal validity. Apart from one study that recruited more than 400 participants, the sample size of the other studies was ≤20, and of six of them was <10, which was far from sufficient, particularly for those applied machine learning models (vulnerable to under-fitting). Data were normally augmented or pooled on the participants by repeating trials or multiple epoch samples from the full record. Besides, gender could also be a significant confounder because of the larger Adam’s apple in males. We found neither stratified analysis nor feature input using gender. 

For internal validity, most studies were prone to selection bias with imbalanced classes, which could be observed by the disagreement among sensitivity, specificity and PPV. Classification of an Imbalanced dataset (uneven class distribution) is among one of the most pervasive fallacies in the field [44]. For epoch-based classification, people spent substantially more non-swallowing time than swallowing time in a given period. One may make a correct guess on non-swallowing events simply by chance, which explains the high specificity (classifying non-swallowing correct most of the time, therefore a high number of true negatives) but low sensitivity and PPV (a large number of false positives) in some studies. Nevertheless, Lee et al. [36] attempted to resolve the imbalanced class problem with a bootstrapping approach. Another source of the imbalanced class could be due to the imbalanced fine-grained classification. There could only be one class of non-swallowing event (saliva swallowing) but multiple classes of swallowing events (e.g., eating different kinds of food). Besides, several studies discarded some data because of noise or corruption, which constituted to selection and attrition biases.

Protocol heterogeneity may hinder the translational potential of wearable technology in this field. The International Dysphagia Diet Standardisation Initiative (IDDSI) framework provides a set of descriptions and definitions on the levels of food textures and drink thickness, which may help in unifying the assessment tasks [45]. Nevertheless, non-swallowing events and dry swallows are not included in the IDDSI framework. From this review, we noticed that existing studies attempted to classify non-vocal and vocal activities. Non-vocal activities included gargling, throat clearing, yawning, and sniffing, while vocal activities included coughing, humming, and reading (pronouncing vowels).

There were some limitations in this study. The inclusion criterion on publications in English may lead to language bias in our review, while selection bias may happen since the searched databases may not include conference abstracts or other types of publications. Due to the heterogeneity of the studies in protocols, event stamps (epoch-based versus event-based), and classes (swallowing versus non-swallowing, and classification of different food), the definitions of performance metrics could be different, which was further complicated by the attrition bias and imbalanced class size. Therefore, we are not confident in comparing and concluding how different types of sensors, feature extraction strategies and classifiers impact the performance. Moreover, it shall be noted that a high classification accuracy in identifying specific swallow events might not manifest that the protocols or chosen swallow events are clinically adequate or relevant to broader applications, such as screening for dysphagia.

In terms of the scope, we did not include relevant research on dysphagia, post-stroke, and aspiration individuals in our review, considering that the current state-of-the-art might not even be sufficient to accurately recognize a “healthy” swallow event. In fact, there were already some studies that applied the techniques to screen non-healthy swallows. Khalifa et al. [43] proposed and validated an automatic swallowing event extraction algorithm to segment the physiological signature of the swallowing process for stroke patients. Steele et al. [46] developed a signal processing classifier using linear discriminant analysis to predict impaired swallowing from patients at-risk, including those with stroke and brain injury. Besides, there were also other types of wearable sensors not within the scope of this review, such as EMG, ultrasound, and biomaterials (e.g., flexible biosensors). Shieh et al. [47] integrated sEMG, nasal airflow sensor and force sensing resistor to quantify the swallowing functions. Hashimoto et al. [48] made use of a Kinect sensor to trace the biomotion of the laryngeal region and successfully segmented the swallowing process from the oral to the laryngeal phase. Using ultrasonography, Matsuo and Matsuyama [49] visualized the hyoid bone and larynx movement in an attempt to identify the contributing factor to dysphagia. Besides, several studies applied biomaterials, such as hydrogels, nanofiber membranes, and carbon nanotubes over the throat, to detect throat motions [50,51,52].

In fact, wearable sensors using accelerometers may cause discomfort and lead to non-compliance issues, especially in older adults with dementia [53] that commonly co-occur with dysphagia [54]. The behavioral activity of the older adults would also affect the accuracy and induced noise to the swallowing accelerometric signal [55]. For acoustics sensors, most of the studies in the review controlled the noise level during the experiment, while some discarded the data that were polluted by noise, which led to concerns about the practicability of the system in real practice. It is pragmatically demanding to improve the current wearable technology in accurate swallow detection and therefore screening for dysphagia and other swallowing disorders. Future studies may also consider transforming the sensors to biofeedback or controllers for virtual reality and gamified swallowing therapy [56,57]. 

## 5. Conclusions

Current wearable technology using acoustics-based or/and accelerometric-based sensors could not achieve adequate accuracy in recognizing swallowing events in general. The studies were also prone to bias because of the small sample size and imbalanced class size. The high heterogeneity of the studies called for a standardized assessment protocol that could account for swallowing, dry swallowing, and non-swallowing tasks. Besides, there is a need to improve the current wearable technology and the credibility of relevant research for accurate swallowing detection before translating into clinical screening for dysphagia and other swallowing disorders.

## Figures and Tables

**Table 1:** Instrument setting and location in the reviewed articles.

Author (Year)	Sensors	Location
Afkari [30]	Miniature ACC (NM)	Level of thyroid cartilage
	sEMG (NM)	Level of cricopharyngeus muscle
	Omnidirectional electret MIC (NM)	Level of cricoid cartilage
Amft and Troster [31]	sEMG (Nexus-10, MindMedia)	Collar at infra-hyoid throat region
	Stethoscope MIC (ECM-C115, Sony)	Collar below hyoid
Bi et al. [32]	Throat MIC [NM]	Over neck close to the jaw
Fontana et al. [33]	Condenser MIC (CZN-15E)	thyroid cartilage level, one side of the neck
	Piezoelectric MIC (IASUS NT, IASUS Concept Ltd.)	Over laryngopharynx
Fukuike et al. [34]	Condenser MIC (WM-61A, Panasonic, Osaka, Japan)	Fixed on a silicone tube and placed inside the left nostril
	Laryngeal MIC (SH-12iK, Nanzu, Shizuoka, Japan)	Over anterior larynx
Kurihara et al. [35]	Bi-directional electret condenser MIC (EM114, Primo Co., Ltd.)	MIC attached to air tube hung over neck with anterior opening
Lee et al. [36]	Dual axis ACC (ADXL322)	Below thyroid cartilage aligned in anterior-posterior and superior-inferior axes
	Submental mechanomyography (developed by Silva and Chau [42])	On the geniohyoid
	Pressure Transducer (PTAFLITE, Glass Technologies)	At nasal cannula
Makeyev et al. [37]	Throat microphone (IASUS NT, IASUS Concept Ltd.) *	Over laryngopharynx
Sazonov et al. [38]	Throat microphone (IASUS NT, IASUS Concept Ltd.) *	Over laryngopharynx
Sejdic et al. [39]	Dual-axis accelerometer (ADXL322)	Anterior to cricoid cartilage, along anterior-posterior and superior-inferior axes
Skowronski et al. [40]	Miniature surface-mounted MIC (VT506, Voice Technologies, Zurich, Switzerland)	Laterally below the cricoid cartilage

ACC: accelerometer; MIC: microphone; NM: Source not mentioned; and sEMG: surface electromyography. * Articles mentioned that it is a multimodal system, but other modalities were not presented.

**Table 2:** Protocol and Procedure for Swallowing Assessment or Detection.

Author (Year)	Subject	Class	Procedure	Protocol
Afkari [30]	1	sw vs. nsw	sw: drink 100 mL of water as fast as possible nsw: dry (saliva) swallowing	Four 30-min sessions performing 3 dry & one swallow
Amft and Troster [31]	4M/2F	sw vs. nsw	Participants were allowed to move, chew, & speak normally during the recording. The participants were asked to drink 5 mL & 15 mL of water, eat a spoonful of yogurt, & 2 cm^3^ of bread in one piece	2 intake sessions on different days
Bi et al. [32]	5F/7M	Solid vs. liquid; food type	Apple, carrot, chip, cookie, peanut, walnut, water	Food was excluded if participants disliked it. Total 560 events
Fontana et al. [33]	7	food type	Start with 5 min quiet sitting 5 min reading aloud a meal of 4 food items (apple, 40 g crackers, low-fact yogurt, 250 mL water) was consumed at unlimited time	10 repetitions for each food in a single swallow with 20 s of talking time between food intake
Fukuike et al. [34]	4F/3M	sw vs. nsw	sw: taking a meal and stepping on a foot pedal when swallowed nsw: yawn, cough, sigh, throat clearing, gargling, and sipping tea	-
Kurihara et al. [35]	7M	sw (food type) vs. nsw	sw: tea (10 mL), tea with a thickener (10 mL), rice cake (10 g) nsw: swallowing nothing	10 repetitions
Lee et al. [36]	8M/9F	sw vs. nsw	Water, barium suspension (Ba), nectar-thick apple juice (Ne), honey-thick apple juice (Ho), spoon-thick apple juice (Sp)	Except for Sp, other drinks involved discrete and continuous tasks. Each task was repeated twice. Water was repeated 3 times
Makeyev et al. [37]	12	sw vs. nsw	Start with 10 min silent 10 min reading aloud Meal of fixed size consumed at an unlimited time (including cheese pizza, yogurt, apple, peanut butter sandwich) 10 min silent 10 min reading aloud	4 visits
Sazonov et al. [38]	20	sw vs. nsw	20 min rest A meal 20 min rest	4 visits
Sejdic et al. [39]	408	sw vs. nsw (head position)	nsw: dry (saliva) swallow sw: drink water in natural & chin-tucked position	5 swallows for each condition
Skowronski et al. [40]	9	sw vs. nsw (type)	sw: 5 mL liquid nsw: dry swallow, head move, yawn, sniff, tongue move, speech, hum, throat clear, cough	10 repetitions

nsw: non-swallowing; sw: swallowing; vs: versus.

**Table 3:** Segmentation and Feature Extraction Strategies.

Author (Year)	Event Stamp	Segmentation Methods	Feature Extraction Strategy/Source
Afkari [30]	Ev	Manual segmentation	Time domain raw signal
Amft and Troster [31]	Ep	Frame at 250 ms	Feature Similarity Instance
Bi et al. [32]	Ev	HMM-based on Mel frequency cepstral coefficients	Predetermined time-domain, frequency-domain, and non-linear features
Fontana et al. [33]	Ep	Frame at 1.0 s & 1.5 s	Time domain raw signal
Fukuike et al. [34]	Ev	Identifying the semblable wave period by moving average. A period longer than 0.35 s was regarded as a swallowing event	Time domain raw signal
Kurihara et al. [35]	Ev	Manual prepared template for pattern matching	
Lee et al. [36]	Ep	Frame at 200 ms with 50% overlap	Signal variance
Makeyev et al. [37]	Ev & Ep	1.5 s epoch	Mel-scale Fourier spectrum with PCA
Sazonov et al. [38]	Ev & Ep	Grid search on epoch duration and step size	Frequency domain raw signal
Sejdic et al. [39]	Ev	Minimum Description Length-based Sequential Segmentation	Time domain raw signal
Skowronski et al. [40]	Ep	Manually segmentation at 6 s	Human factor cepstral coefficients and spectral flatness measure

Ep: Epoch-based; Ev: Event-based; HMM: Hidden Markov Model; PCA: Principal Component Analysis.

**Table 4:** Classification performance for swallow detection or classification.

Author (Year)	Classifier	Precision/PPV	Recall/Sensitivity	Specificity	Accuracy
Afkari [30]	TB	-	-	-	dry swallow 94.3% swallow: 92.75%
Amft and Troster [31]	LR	10%	65%	-	-
	AGREE	20%	68%	-	-
Bi et al. [32]	HMM (Event)	-	-	-	86.6%
	DT	86.2%	87.5%	-	87.1%
Fontana et al. [33]	TB	50.1%	86.1%	-	68.2%
Fukuike et al. [34]	TB	-	97.2%	95.2	-
Kurihara et al. [35]	Template matching	-	-	-	88.8% *
Lee et al. [36]	ANN	-	91%	88.2%	88.5%
Makeyev et al. [37]	SVM (Epoch)	-	44%	99%	95.7%
	SVM (Event)	-	71.3%	87%	80.4%
Sazonov et al. [38]	SVM (Epoch)	-	-	-	96.4%
	SVM (Event)	-	-	-	96.8%
Sejdic et al. [39]	2-class fuzzy c-means	-	-	-	94.6%
Skowronski et al. [40]	GMM	-	89.5%	98%	96.3%

AGREE: Agreement Fusion of detectors; DT: Decision Tree; TB: Threshold-based; GMM: Gaussian Mixture Model; HMM: Hidden Markov Model; LR: Logistic Regression; SVM: Support Vector Machine. * Accuracy was calculated by the weighted average of class accuracy.

## Data Availability

Not applicable.
